# HIV prevalence and factors associated with HIV infection among men who have sex with men in Cameroon

**DOI:** 10.7448/IAS.16.4.18752

**Published:** 2013-12-02

**Authors:** Ju Nyeong Park, Erin Papworth, Sethson Kassegne, Laure Moukam, Serge Clotaire Billong, Issac Macauley, Yves Roger Yomb, Nathalie Nkoume, Valentin Mondoleba, Jules Eloundou, Matthew LeBreton, Ubald Tamoufe, Ashley Grosso, Stefan D Baral

**Affiliations:** 1 Center for Public Health and Human Rights, Department of Epidemiology, Johns Hopkins Bloomberg School of Public Health, Baltimore, MD, USA; 2 West and Central Africa Region, Population Services International, Cotonou, Benin; 3 Association Camerounaise pour le Marketing Social (ACMS), Yaoundé, Cameroon; 4 Comité national de lutte contre le sida (CNLS), Ministère de la Sante Publique (MINSANTE), Yaoundé, Cameroon; 5 CARE International-Cameroon, Yaoundé, Cameroon; 6 Alternatives-Cameroun, Douala, Cameroon; 7 CAMNAFAW, Yaoundé, Cameroon; 8 Humanity First, Yaoundé, Cameroon; 9 Global Viral Cameroon, Yaoundé, Cameroon

**Keywords:** Men who have sex with men (MSM), HIV/AIDS, epidemiology, Africa, prevalence, respondent-driven sampling (RDS), homosexuality, prevention, risk factors, sexual behaviour

## Abstract

**Introduction:**

Despite men who have sex with men (MSM) being a key population for HIV programming globally, HIV epidemiologic data on MSM in Central Africa are sparse. We measured HIV and syphilis prevalence and the factors associated with HIV infection among MSM in Cameroon.

**Methods:**

Two hundred and seventy-two and 239 MSM aged ≥18 from Douala and Yaoundé, respectively, were recruited using respondent-driven sampling (RDS) for this cross-sectional surveillance study in 2011. Participants completed a structured questionnaire and HIV and syphilis testing. Statistical analyses, including RDS-weighted proportions, bootstrapped confidence intervals and logistic regressions, were used.

**Results:**

Crude and RDS-weighted HIV prevalence were 28.6% (73/255) and 25.5% (95% CI 19.1–31.9) in Douala, and 47.3% (98/207) and 44.4% (95% CI 35.7–53.2) in Yaoundé. Active syphilis prevalence in total was 0.4% (2/511). Overall, median age was 24 years, 62% (317/511) of MSM identified as bisexual and 28.6% (144/511) identified as gay. Inconsistent condom use with regular male partners (64.1%; 273/426) and casual male and female partners (48.5%; 195/402) was common, as was the inconsistent use of condom-compatible lubricants (CCLs) (26.3%; 124/472). In Douala, preferring a receptive sexual role was associated with prevalent HIV infection [adjusted odds ratio (aOR) 2.33, 95% CI 1.02–5.32]. Compared to MSM without HIV infection, MSM living with HIV were more likely to have ever accessed a health service targeting MSM in Douala (aOR 4.88, 95% CI 1.63–14.63). In Yaoundé, MSM living with HIV were more likely to use CCLs (aOR 2.44, 95% CI 1.19–4.97).

**Conclusions:**

High HIV prevalence were observed and condoms and CCLs were used inconsistently indicating that MSM are a priority population for HIV prevention, treatment and care services in Douala and Yaoundé. Building the capacity of MSM community organizations and improving the delivery and scale-up of multimodal interventions for MSM that are sensitive to concerns about confidentiality and the complex individual, social, community-level and policy challenges are needed to successfully engage young MSM in the continuum of HIV care. In addition to scaling up condom and CCL access, evaluating the feasibility of novel biomedical interventions, including antiretroviral pre-exposure prophylaxis and early antiretroviral therapy for MSM living with HIV in Cameroon, is also warranted.

## Introduction

Globally, it has been observed that HIV prevalence among men who have sex with men (MSM) significantly exceeds HIV prevalence in the general population, even in the context of generalized epidemics [1–3]. Across sub-Saharan Africa, HIV prevalence is estimated to be approximately 5% in the general population and 17.9% among MSM [1]. The few published studies from West Africa consistently report higher HIV prevalence among MSM than in the general population, with HIV prevalence estimates of 13.5% among MSM in Nigeria, 16.3% in Burkina Faso and 21.8% in Senegal [1,2,4–6]. Individual-, network-, community-level and policy-level factors noted to contribute to the higher risk of acquisition and transmission of HIV and other sexually transmitted infections (STIs) among MSM have been found to be prevalent in Central and West Africa [5,7,8].

With over 550,000 people living with HIV in Cameroon, the prevalence of HIV among reproductive-age adults in Cameroon is estimated to be 4.3%, which represents a mature and widespread generalized epidemic [9,10]. In Douala and Yaoundé, the two largest cities of the country, HIV prevalence among reproductive-age adults is estimated to be 4.6% and 6.3%, respectively [10].

MSM were recently listed as a priority group in the Cameroon government's “National Strategic Plan for HIV, AIDS, and STIs: 2011–2015,” along with goals including strengthening HIV-prevention programmes and building capacity for HIV health services that serve MSM [11]. The higher biological risks of HIV acquisition and transmission associated with unprotected anal intercourse (UAI) compared to other forms of sexual intercourse make MSM an important target population for HIV-prevention efforts [12]. However, only one HIV prevalence estimate from programmatic data in Douala is available to date for MSM; in this 2007 study, which used convenience sampling, HIV prevalence was estimated to be 18.4% [13].

Established individual-level risks for HIV acquisition and transmission among MSM in the region that are modifiable include UAI, inconsistent use of condom-compatible lubricants (CCLs), a high number of male partners, drug use and syphilis co-infection [1]. In a recent study, UAI in the past 6 months was frequent among MSM in Douala, as was having one or more female sexual partners [7]. Bisexual concurrency and bisexual partnerships among MSM have been observed in studies in Nigeria, Senegal and southern Africa [5,14,15]. Inconsistent condom use with male and female partners was common among MSM in one Togo study, and in a study conducted in Nigeria, it was associated with prevalent HIV infection, as was having been the receptive partner in anal intercourse in the past 6 months [5,16]. Other factors associated with prevalent HIV infection among MSM in Nigeria and Senegal were older age and having a symptomatic STI [5,15].

Network-level factors that may impact HIV-transmission risk include sexual network size, STI prevalence, levels of peer education, knowledge of HIV status within the population and network tendencies for drug use or transactional sex [1]. Community-level factors that may contribute to HIV risk include high community viral load and suboptimal coverage or uptake of healthcare services [1]. Additionally, the social stigma surrounding HIV, sexual identities and homosexuality in Cameroon may deter MSM from seeking voluntary HIV counselling and testing (VCT) or other health services [17–20]. Perceived stigma, including fear of seeking healthcare and refraining from disclosing same-sex practices to a health professional, and enacted discrimination, including denial of healthcare access based on sexuality, were frequently reported by MSM in Senegal and southern Africa, and were associated with increased sexual risk practices and prevalent HIV infection [21–26]. Similar to most countries in sub-Saharan Africa, sexual relationships between men are both criminalized and highly stigmatized in Cameroon, and prosecution can result in up to 5 years of imprisonment [8]; physical violence from law enforcement is also a reality for some MSM, posing challenges to HIV programming [8,27–29].

In light of the unique needs of MSM within generalized epidemics, and the limited data available on this vulnerable population in Cameroon, we aimed to describe the socio-demographic and behavioural characteristics of MSM in Douala and Yaoundé, determine the age-stratified HIV and syphilis prevalence in both cities, and investigate the individual-, network- and community-level factors associated with HIV infection among this population.

## Methods

### Study population

This cross-sectional study was conducted in August–September 2011 at two community-based organizations (CBOs) that provide targeted services to MSM: Alternatives-Cameroun in Douala and the Cameroon National Association for Family Welfare (CAMNAFAW) in Yaoundé. The interviewers were MSM community volunteers from Alternatives-Cameroun, Humanity First and CAMNAFAW. The MSM sensitivity trainings for interviewers were conducted at the Association Camerounaise pour le Marketing Social (ACMS) conference rooms in Douala and Yaoundé. Men aged 18 years or older who reported engaging in penile–anal or oral intercourse with another man in the past 12 months were eligible for the study. Participants were recruited using respondent-driven sampling (RDS) [30], a sampling technique that enables estimation of proportions and regression modelling while controlling for non-random social network structures that bias peer-based recruitment. Seven seeds heterogeneous in sexual identity and sexual role preference were selected through existing community contacts to begin the recruitment process in each city. Upon enrolment in the study, all individuals were given three uniquely coded coupons to refer other MSM to the study. The CBOs worked with the research team to identify the initial seeds, screen study participants for eligibility and interview participants after receiving informed consent.

Sample-size calculations were based on the ability to detect a 15% change in the prevalence of condom use at last anal intercourse over time from 60% at baseline, with a design effect of 2, a significance level of 0.05 and a power of 80%, yielding 241 men for each city.

All participants provided written informed consent. The study was approved by the Cameroon National Ethics Committee, and the secondary analysis of the study data was approved by the Johns Hopkins Bloomberg School of Public Health.

### Data collection

Participants completed an interviewer-administered structured questionnaire containing questions on: socio-demographics; network size; sexual behaviours, including condom and lubricant use (always vs. often, sometimes or never); experiences of STI symptoms; access to CBO-run MSM centres (which included outreach services); access to free condoms; VCT experiences; knowledge of HIV transmission, prevention and treatment (a composite score from 13 questions); and perceived social support for condom use (a composite score from eight questions, including support from partners, family and peers). Interviews were conducted in French or English, and they were recorded in French.

After participants received pre-test counselling, approximately 4 ml blood specimen was collected from them by a Global Viral Cameroon phlebotomist and tested to confirm HIV and syphilis serostatus, followed by post-test counselling on the same day. Men who screened positive for HIV or syphilis were referred to appropriate health services. All participants were reimbursed 1000 CFA franc (US$2) for completing the questionnaire and an additional 1000 CFA franc (US$2) for each peer referred into the study. All participants received free VCT, condoms and CCLs. Participants were also given access to peer education, support groups and linkage to HIV care.

### Laboratory testing

Specimen processing and testing were conducted by staff from Global Viral Cameroon at the field sites. The national HIV surveillance algorithm for second-generation surveillance of HIV, adopted by the Ministry of Public Health of Cameroon, was used to measure current HIV status, including Determine^®^ HIV-1/2 (Inverness Medical, Chiba, Japan) and Human HEXAGON HIV 1 + 2 (Human GmBh, Wiesbaden, Germany). All indeterminate and positive samples and 15% of the negative samples were transferred to the Global Viral Cameroon Yaoundé laboratory for fourth-generation HIV enzyme-linked immunosorbent assay (ELISA), which detects antibodies to HIV-1/2 and the p24 antigen (whose presence indicates a possible seroconversion). Screening for syphilis was performed according to the national algorithm in Cameroon using Rapid Protein Reagin (RPR; SGM Italia, Roma, Italy) and *Treponema pallidum* haemagglutination assay (TPHA; Fortress Diagnostics Limited, Antrim, UK). Global Viral Cameroon was responsible for blood specimen collection, laboratory testing and serology data management.

### Statistical analysis

ACMS and CARE International-Cameroon managed study data. Questionnaire data were double entered into the CSPro (version 4.0) software, exported into SPSS for data cleaning by ACMS and then exported to Stata/SE (version 11.2) for data analysis.

To minimize biases associated with chain referral sampling, weights were created in Stata/SE version 11.2 using the RDSII estimator to account for the effect of differences in the social network sizes of participants. Weights were based on the transition matrix for the dependent variable, current HIV status. Network size was assessed using the response from the latter of two questions: “How many men who have had oral or anal sex with men in the last 12 months do you know, who also know you and live in this city?” and “among these men that you know personally, how many of them are 18 years and older?” Homophily (range: −1 to +1) was assessed to evaluate the preferences of individuals to recruit MSM with the same HIV status [31].

Bivariate logistic regression models were used to estimate the unadjusted association between HIV infection and covariates selected based on our knowledge and the published literature. RDS-weighted prevalence and bootstrapped confidence intervals were calculated for all variables explored in regression modelling. Multivariate logistic regression models were built to estimate the adjusted association between current HIV status and covariates, with age forced into all models regardless of statistical significance. The Akaike information criterion (AIC) was used to favour the most parsimonious models. Bivariate and multivariate logistic regression models were also built with RDS weighting. *p*-values < 0.05 were used to indicate statistical significance. We further compared the associations between binary covariates using the Pearson chi-square test.

## Results

A total of 295 men were screened in Douala, of whom 272 participated. In Yaoundé, a total of 246 individuals were screened, resulting in 239 participants. The median number of descendants per seed was 32 (range 6–99) in Douala and 31 (range 2–88) in Yaoundé. In Douala, the median number of waves per seed was 6 (range 1–8); homophily for HIV status was −0.04 among the HIV-negative group and 0.06 in the group living with HIV. In Yaoundé, the median number of waves per seed was five (range 1–9); homophily for HIV status was 0.004 for the HIV-negative group and 0.06 for the group living with HIV. In both samples, RDS network homophily was close to 0, which may indicate a close approximation to random recruitment. The majority (77.9%; 398/511) reported that they would have given a coupon to their recruiter (an indicator of the reciprocal ties assumption [32]).

Overall, the median age was 24 years (range 18–51, interquartile range (IQR) 21–28). In both cities, the majority had completed secondary education and were single. Sixty-two percent of MSM in the overall sample identified as bisexual, compared with 28.6% who identified as gay or homosexual and 9.8% as MSM or other. Ninety-eight percent of all participants reported having penile-anal intercourse in the past 12 months. Median age of sexual debut with another man was 19 (IQR 17–22) (Table 1).

**Table 1:** Characteristics of MSM recruited from Douala (*n*=272) and Yaoundé (*n*=239) in Cameroon, 2011

		Douala		Yaoundé	
			
	All *n* (%)	*n* (%)	RDS-weighted % (95% CI)	*n* (%)	RDS-weighted % (95% CI)
Total	511 (100)	272 (100)	–	239 (100)	–
Age, median (IQR) (years)	24 (21–28)	23 (21–27)	–	25 (21–28)	–
18–23	238 (46.6)	142 (52.2)	57.6 (50.6–64.6)	96 (40.2)	42.1 (34.0–50.3)
24–29	185 (36.2)	85 (31.3)	29.5 (23.1–35.9)	100 (41.8)	42.5 (34.7–50.3)
30+	88 (17.2)	45 (16.5)	12.9 (8.2–17.6)	43 (18.0)	15.3 (8.9–21.8)
Education					
Primary or less	26 (5.1)	20 (7.4)	7.6 (4.0–11.2)	6 (2.5)	2.7 (0.1–5.2)
Secondary	341 (66.7)	183 (67.3)	70.1 (64.1–76.1)	158 (66.1)	69.8 (63.5–76.2)
Higher than secondary	144 (28.2)	69 (25.8)	22.3 (16.5–28.0)	75 (31.5)	27.5 (21.5–33.6)
Occupational status					
Student or apprentice	204 (39.9)	116 (42.7)	46.5 (40.0–53.0)	88 (36.8)	36.9 (29.3–44.5)
Employed	248 (48.5)	126 (46.3)	45.0 (38.1–51.8)	122 (51.1)	48.8 (41.0–56.6)
Unemployed	59 (11.6)	30 (11.0)	9.1 (5.3–12.9)	29 (12.1)	14.3 (7.7–20.9)
Christian religion	456 (89.2)	231 (86.2)	87.9 (83.2–92.6)	220 (92.4)	91.6 (87.1–96.1)
Network size, median (IQR)	12 (6–25)	13 (5–25)	–	12 (6–24.5)	–
Sexual identity					
Bisexual	317 (62.0)	171 (62.9)	65.9 (59.3–72.5)	146 (61.1)	62.1 (54.1–70.1)
Gay or homosexual	144 (28.6)	70 (26.3)	22.7 (16.8–28.6)	73 (31.3)	28.7 (21.1–36.4)
MSM	41 (8.0)	26 (9.6)	9.4 (5.6–13.2)	15 (6.3)	8.4 (3.5–13.3)
Other	9 (1.8)	4 (1.5)	–	5 (2.1)	–
Relationship status					
Single	425 (84.2)	230 (84.6)	85.6 (80.5–90.6)	194 (83.3)	87.3 (82.2–92.4)
In a relationship or married	77 (15.2)	39 (14.3)	14.4 (9.4–19.5)	38 (15.9)	12.7 (7.6–17.8)
Separated, widowed or other	3 (0.6)	2 (0.8)	–	1 (0.5)	–
Sexual role preference					
Insertive	223 (45.0)	118 (43.9)	45.4 (37.4–53.5)	110 (46.2)	46.8 (38.7–54.9)
Receptive	160 (31.6)	85 (31.6)	29.8 (23.6–36.0)	75 (31.5)	32.2 (24.7–39.8)
Versatile	119 (23.5)	66 (24.5)	24.7 (17.9–31.6)	53 (22.3)	21.0 (15.0–26.9)
Age of sexual debut with another man, median (IQR) (years)	19 (17–22)	19 (17–22)	–	19 (17–21)	–

CI, confidence interval; IQR, interquartile range; MSM, men who have sex with men; RDS, respondent-driven sampling.

Responses to questions on health service uptake, HIV knowledge, social support and sexual practices are presented in Table 2. Men in Yaoundé were much less likely to access CBO services targeting MSM than men in Douala (33.7% vs. 66.1%, χ^2^(1), *p*<0.001). No difference was observed in ever receiving free condoms (74.2% vs. 68.8%, χ^2^(1), *p*=0.2). In both cities, a large proportion of men reported sex with males and females (46.2%) and experienced STI symptoms in the previous year (34.6%). Inconsistent use of condoms with regular male partners (64.1%; 273/426) and casual male and female partners (48.5%; 195/402) was common, as were condom slippage and breakage (43.7%; 216/494). Ninety percent of MSM who used condoms also reported using lubricant. Of these men, 26.3% (124/472) reported using lotion, saliva, Vaseline or other condom-incompatible lubricants.

**Table 2:** Health service uptake, HIV knowledge, social support and sexual practices among MSM recruited from Douala (*n*=272) and Yaoundé (*n*=239) in Cameroon, 2011

		Douala		Yaoundé	
			
	All *n* (%)	*n* (%)	RDS-weighted % (95% CI)	*n* (%)	RDS-weighted % (95% CI)
Ever accessed CBO service targeting MSM	302 (59.1)	199 (74.3)	66.1 (57.6–74.6)	100 (42.0)	33.7 (26.6–40.8)
Ever received free condoms	355 (71.7)	196 (74.2)	71.6 (64.3–78.9)	159 (68.8)	62.2 (54.1–70.4)
HIV knowledge composite, median score % correct (IQR)	85 (77–92)	85 (77–92)	–	85 (77–92)	–
Social support on condom use, median score % (IQR)	63 (38–88)	63 (38–88)	–	75 (50–88)	–
Ever had sexual intercourse after drinking alcohol	338 (66.1)	156 (57.4)	57.5 (49.6–64.8)	182 (76.2)	73.2 (65.9–80.4)
Ever had sexual intercourse after taking a drug	43 (8.4)	18 (6.6)	5.0 (1.7–8.2)	25 (10.5)	9.7 (5.8–13.5)
In the past 12 months					
Had male and female sexual partners	236 (46.2)	125 (46.1)	48.3 (41.2–55.5)	111 (46.4)	49.6 (42.3–56.9)
Experienced STI symptom(s)	175 (34.5)	80 (29.9)	30.5 (23.6–37.4)	95 (39.9)	38.9 (31.5–46.4)
Number of male partners, median (IQR)	3 (2–5)	3 (2–5)	–	3 (2–5)	–
1–3	304 (59.5)	171 (62.9)	68.1 (61.3–74.8)	133 (55.7)	62.9 (55.5–70.3)
4+	207 (40.5)	101 (37.1)	31.9 (25.2–38.7)	106 (44.3)	37.1 (29.8–44.5)
Inconsistent condom use with regular male partner(s)*	273 (64.1)	123 (56.9)	58.4 (49.1–67.8)	100 (42.2)	42.9 (36.3–49.4)
Inconsistent condom use with casual partner(s)**	195 (48.5)	98 (46.9)	36.2 (29.4–43.1)	96 (50.3)	44.4 (33.6–55.2)
Condom torn or removed involuntarily during sex	216 (43.7)	118 (44.9)	46.3 (39.2–53.5)	98 (42.4)	41.6 (34.4–48.7)
Generally use lubricant with condom	460 (90.0)	235 (89.4)	88.5 (83.8–93.2)	219 (92.4)	93.8 (90.6–97.0)
CCLs	348 (73.7)	186 (75.6)	72.2 (65.3–79.0)	162 (71.7)	66.8 (57.9–75.7)
Lotion, saliva, Vaseline or other	124 (26.3)	60 (24.4)	27.8 (20.9–34.8)	64 (28.3)	33.2 (24.3–42.1)
Gave a woman money or objects in exchange for sexual intercourse	25 (4.9)	14 (5.2)	5.7 (1.8–9.7)	11 (4.6)	4.3 (0.8–7.8)
Gave a man money or objects in exchange for sexual intercourse	30 (5.9)	15 (5.5)	4.2 (1.6–6.8)	15 (6.3)	4.4 (1.8–6.9)

* In Douala, *n*=52 (19.4%) did not have a regular partner. In Yaoundé, *n*=32 (13.4%) did not have a regular partner.

** In Douala, *n*=59 (22.0%) did not have a casual partner; in Yaoundé, *n*=48 (20.1%) did not have a casual partner.

CBO, community-based organization; CCLs, condom-compatible lubricant; CI, confidence interval; IQR, interquartile range; MSM, men who have sex with men; RDS, respondent-driven sampling; STI, sexually transmitted infection.

**Figure 1:**
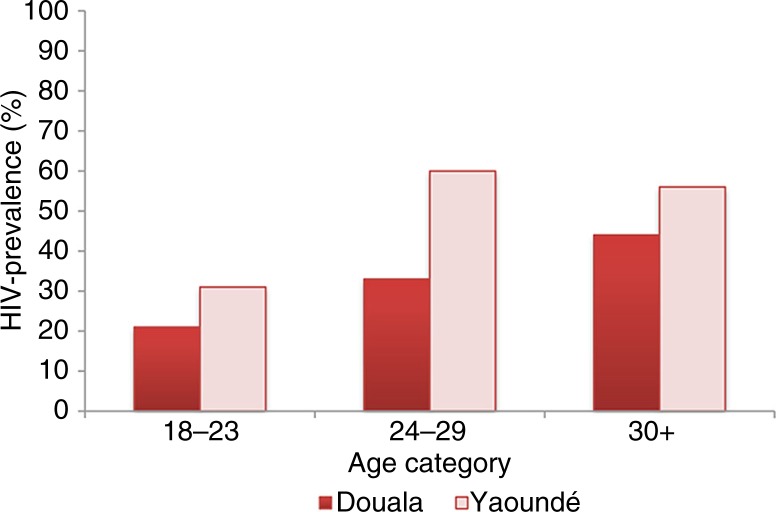
Unadjusted HIV prevalence stratified by age category among MSM from Douala (*n*=255) and Yaoundé (*n*=207) in Cameroon, 2011.

As presented in Table 3, crude and RDS-weighted HIV prevalence were 28.6% (73/255) and 25.5% (95% CI 19.1–31.9) in Douala and 47.3% (98/207) and 44.4% (95% CI 35.7–53.2) in Yaoundé. Age-stratified prevalence is presented in Figure 1. In Douala, only 17 (6.3%) MSM refused to be tested; in Yaoundé, this number was higher (13.4%, *n*=32). An association between having a history of VCT and refusing testing in the study was observed in Yaoundé, although it did not reach statistical significance (15.2% vs. 4.9%, *p*=0.08). Refusal was not correlated with age, education level, age of sexual debut, condom use, receptive sexual role preference, number of male sexual partners in the past 12 months or perceived social support for condom use (all *p*>0.10). Active syphilis infection was detected in only one participant per city.

**Table 3:** HIV and syphilis prevalence among MSM in Douala (*n*=272) and Yaoundé (*n*=239) in Cameroon, 2011

		Douala		Yaoundé	
			
	All *n* (%)	*n* (%)	RDS-weighted % (95% CI)	*n* (%)	RDS-weighted % (95% CI)
HIV prevalence*					
All ages	171 (37.0)	73 (28.6)	25.5 (19.1–31.9)	98 (47.3)	44.4 (35.7–53.2)
Age 18–23	54 (24.9)	27 (20.6)	14.6 (6.7–22.6)	27 (31.4)	20.8 (8.5–33.1)
Age 24–29	79 (47.0)	27 (33.3)	30.0 (16.9–43.1)	52 (60.0)	59.7 (48.8–70.6)
Age 30+	38 (49.4)	19 (44.2)	43.8 (27.9–59.7)	19 (55.9)	55.9 (29.7–82.1)
Refused HIV testing	49 (9.6)	17 (6.3)	4.2 (1.8–6.6)	32 (13.4)	12.7 (7.3–18.2)
Active syphilis infection**	2 (0.4)	1 (0.5)	–	1 (0.4)	–

* HIV status determined by two rapid tests, and confirmation by enzyme-linked immunosorbent assay (ELISA) test.

** Positive in both Rapid Protein Reagin (RPR) and *Treponema pallidum* hemagglutination assay (TPHA) tests.

CI, confidence interval; MSM, men who have sex with men; RDS, respondent-driven sampling.

### Factors associated with HIV infection

#### Douala

Bivariate and multivariate analyses of the individual-, network- and community-level factors associated with HIV infection in Douala are presented in Table 4. The adjusted RDS-weighted odds of having HIV increased for every year rise in age for MSM aged 18–29 (aOR 1.13 per year, 95% CI 1.01–1.27), then plateaued for MSM aged 30 and older (aOR 0.89 per year, 95% CI 0.72–1.09). Preference for the receptive sexual role (aOR 2.33, 95% CI 1.02–5.32) was associated with increased odds of having HIV in both RDS-naïve and RDS-weighted multivariate analyses. Individuals living with HIV were more likely to have ever accessed a CBO service for MSM compared to individuals without HIV (aOR 4.88, 95% CI 1.63–14.63). Individuals who had sex with women (OR 0.50, 95% CI 0.26–0.96) or used condoms inconsistently with casual partners (OR 0.40, 95% CI 0.18–0.89) in the past 12 months were less likely to be living with HIV in bivariate analysis.

**Table 4:** Bivariate and multivariate models of the individual-, network- and community-level factors associated with HIV infection among MSM in Douala (*n*=255) in Cameroon, 2011

	Living with HIV (*n*=73)	HIV-negative (*n*=182)	OR (95% CI)	RDS-weighted OR (95% CI)	aOR (95% CI)	*p*	RDS-weighted aOR (95% CI)	*p*
Age								
Per-year increase for MSM aged 18–29	54 (74.0)	158 (86.8)	**1.13 (1.04–1.23)**	**1.14 (1.04–1.25)**	**1.15 (1.04–1.27)**	**0.005**	**1.13 (1.01–1.27)**	**0.03**
Per-year increase for MSM aged 30+	19 (26.0)	24 (13.2)	0.86 (0.73–1.01)	0.86 (0.72–1.03)	0.86 (0.71–1.04)	0.1	0.89 (0.72–1.09)	0.3
Education: higher than secondary	20 (27.4)	44 (24.2)	1.18 (0.64–2.19)	1.05 (0.52–2.14)	–	–	–	–
Occupational status								
Student or apprentice	17 (23.3)	92 (50.6)	Ref	Ref	–	–	–	–
Employed	47 (64.4)	74 (40.7)	**3.44 (1.82–6.48)**	**3.66 (1.90–7.04)**	–	–	–	–
Unemployed	9 (12.3)	16 (8.8)	**3.04 (1.16–8.00)**	2.09 (0.72–6.10)	–	–	–	–
Christian religion	64 (87.7)	160 (87.9)	0.98 (0.43–2.24)	1.05 (0.40–2.72)	–	–	–	–
Sexual identity: gay	29 (39.7)	39 (21.4)	**2.42 (1.34–4.35)**	**2.53 (1.27–5.03)**				
Relationship status: single	54 (75.0)	160 (87.9)	**0.41 (0.21–0.83)**	0.48 (0.21–1.08)	–	–	–	–
Sexual role preference: receptive	33 (45.2)	48 (26.4)	**2.30 (1.31–4.06)**	**2.23 (1.15–4.31)**	**2.96 (1.50–5.82)**	**0.002**	**2.33 (1.02–5.32)**	**0.045**
Age of sexual debut								
5–17	28 (38.4)	62 (34.1)	Ref	Ref	–	–	–	–
18+	45 (61.6)	120 (65.9)	0.83 (0.47–1.46)	0.66 (0.35–1.26)	–	–	–	–
Ever accessed CBO service targeting MSM	65 (89.0)	125 (68.7)	**3.71 (1.67–8.23)**	**4.33 (1.75–10.75)**	**3.22 (1.17–8.89)**	**0.048**	**4.88 (1.63–14.63)**	**0.005**
Ever received free condoms	60 (84.5)	124 (70.1)	**2.33 (1.14–4.78)**	**2.82 (1.22–6.53)**	–	–	–	–
Generally use CCLs with condoms	59 (81.9)	113 (62.8)	**2.69 (1.37–5.27)**	**2.89 (1.17–7.16)**	**2.32 (1.01–5.34)**	**0.049**	2.29 (0.95–5.53)	0.07
HIV knowledge composite score, per 20% increase	85 (8)	85 (23)	0.85 (0.56–1.29)	0.81 (0.49–1.33)	–	–	–	–
Social support composite score, per 20% increase	75 (50)	63 (50)	1.06 (0.88–1.29)	1.11 (0.88–1.40)	–	–	–	–
In the past 12 months								
Had male and female sexual partners	26 (35.6)	89 (48.9)	0.58 (0.33–1.01)	**0.50 (0.26–0.96)**	–	–	–	–
Any STI symptom	26 (36.1)	49 (27.4)	1.50 (0.84–2.68)	1.38 (0.70–2.74)	–	–	–	–
Number of male partners								
1–3	46 (63.0)	116 (63.7)	Ref	Ref	–	–	–	–
4+	27 (37.0)	66 (36.3)	1.03 (0.59–1.81)	1.40 (0.72–2.70)	–	–	–	–
Inconsistent condom use: regular male partner(s)	36 (60.0)	79 (54.9)	1.23 (0.67–2.28)	1.30 (0.64–2.66)	–	–	–	–
Inconsistent condom use: casual partner(s)	15 (28.9)	76 (52.4)	**0.37 (0.18–0.73)**	**0.40 (0.18–0.89)**	–	–	–	–
Condom slippage or breakage	77 (44.0)	34 (47.2)	1.14 (0.66–1.97)	1.28 (0.68–2.44)	–	–	–	–

Bold indicates *p*-value<0.05. aOR, adjusted odds ratio; CBO, community-based organization; CCL, condom-compatible lubricant; CI, confidence interval; IQR, interquartile range; MSM, men who have sex with men; OR, odds ratio; RDS, respondent-driven sampling; STI, sexually transmitted infection.

#### Yaoundé

In multivariate analysis of the Yaoundé sample (Table 5), factors independently associated with having HIV infection were age (aOR 1.14, 95% CI 1.02–1.26 if aged 18–29; aOR 0.84, 95% CI 0.65–1.07 if aged ≥30) and general use of CCLs with condoms (aOR 2.44, 95% CI 1.19–4.97). Men living with HIV were more likely to have four or more partners in the past 12 months, although this did not reach statistical significance (aOR: 1.88, 95% CI: 0.95–3.71).

**Table 5:** Bivariate and multivariate models of the individual-, network- and community-level factors associated with HIV infection among MSM in Yaoundé (*n*=207) in Cameroon, 2011

	Living with HIV (*n*=98)	HIV-negative (*n*=109)	OR (95% CI)	RDS-weighted OR (95% CI)	aOR (95% CI)	*p*	RDS-weighted aOR (95% CI)	*p*
Age								
Per-year increase for MSM aged 18–29	79 (80.6)	94 (86.2)	**1.15 (1.05–1.25)**	**1.17 (1.06–1.30)**	**1.11 (1.02–1.22)**	**0.02**	1.14 (1.02–1.26)	**0.02**
Per-year increase for MSM aged 30+	19 (19.4)	15 (13.8)	**0.80 (0.65–0.98)**	**0.79 (0.62–1.00)**	0.84 (0.67–1.04)	0.1	0.84 (0.65–1.07)	0.2
Education: higher than secondary	30 (30.6)	35 (32.1)	0.93 (0.52–1.68)	0.93 (0.48–1.83)	–	–	–	–
Occupational status								
Student or apprentice	28 (28.6)	46 (42.2)	Ref	Ref	–	–	–	–
Employed	53 (54.1)	51 (46.8)	1.71 (0.93–3.13)	1.68 (0.83–3.38)	–	–	–	–
Unemployed	17 (17.4)	12 (11.0)	2.33 (0.97–5.59)	1.88 (0.69–5.11)	–	–	–	–
Christian religion	92 (93.9)	98 (89.9)	1.72 (0.61–4.84)	1.23 (0.38–3.97)	–	–	–	–
Sexual identity: gay	38 (38.8)	27 (24.8)	**1.92 (1.06–3.49)**	**2.36 (1.19–4.68)**	–	–	–	–
Relationship status: single	83 (85.6)	92 (87.6)	0.84 (0.37–1.89)	1.36 (0.54–3.46)	–	–	–	–
Sexual role preference: receptive	35 (35.7)	32 (29.4)	1.34 (0.75–2.40)	1.35 (0.69–2.62)	–	–	–	–
Age of sexual debut								
5–17	34 (34.7)	31 (28.4)	Ref	Ref	–	–	–	–
18+	64 (65.3)	78 (71.6)	0.75 (0.42–1.35)	0.67 (0.34–1.31)	–	–	–	–
Ever accessed CBO service targeting MSM	43 (43.9)	44 (40.4)	1.15 (0.66–2.01)	0.95 (0.51–1.78)	–	–	–	–
Ever received free condoms	70 (72.2)	68 (66.7)	1.30 (0.71–2.38)	1.25 (0.62–2.49)	–	–	–	–
Generally use CCLs with condoms	74 (76.3)	59 (54.6)	**2.67 (1.46–4.88)**	**2.42 (1.19–4.91)**	**1.97 (1.04–3.72)**	**0.04**	**2.44 (1.19–4.97)**	**0.02**
HIV knowledge composite score, per 20% increase	85 (15)	85 (15)	0.90 (0.58–1.40)	1.00 (0.60–1.69)	–	–	–	–
Social support composite score, per 20% increase	75 (38)	63 (38)	1.14 (0.91–1.43)	1.18 (0.91–1.53)	–	–	–	–
In the past 12 months					–	–	–	–
Had male and female sexual partners	41 (41.8)	52 (47.1)	0.79 (0.45–1.37)	0.63 (0.34–1.18)				
Any STI symptom	48 (49.0)	43 (39.8)	1.45 (0.84–2.52)	1.81 (0.96–3.42)	–	–	–	–
Number of male partners								
1–3	46 (46.9)	71 (65.1)	Ref	Ref	Ref	–	Ref	–
4+	52 (53.1)	38 (34.9)	**2.11 (1.21–3.69)**	**2.25 (1.19–4.28)**	1.81 (0.99–3.28)	0.05	1.88 (0.95–3.71)	0.07
Inconsistent condom use: regular male partner(s)	59 (67.8)	71 (75.5)	0.68 (0.36–1.31)	0.76 (0.36–1.59)	–	–	–	–
Inconsistent condom use: casual partner(s)	43 (56.6)	43 (49.4)	1.33 (0.72–2.47)	1.55 (0.76–3.15)	–	–	–	–
Condom slippage or breakage	46 (45.1)	41 (42.3)	0.89 (0.51–1.56)	0.69 (0.36–1.32)	–	–	–	–

Bold indicates *p*-value<0.05. aOR, adjusted odds ratio; CCL, condom-compatible lubricant; CI, confidence interval; MSM, men who have sex with men; OR, odds ratio; RDS, respondent-driven sampling; STI, sexually transmitted infection.

## Discussion

The high HIV prevalence and inconsistent use of condoms and CCLs observed in this study highlight that MSM are a priority population for HIV prevention, treatment and care services in Douala and Yaoundé. Furthermore, these data suggest that HIV risks are not evenly distributed given the significant differences in HIV prevalence between cities and between MSM sub-populations [1].

The individual-level factors found to be associated with HIV infection indicate that future HIV programming and interventions in Cameroon should address both behavioural and structural hurdles relevant to MSM. Consistent with data from other countries of sub-Saharan Africa [15,16,24], condom breakage and slippage and inconsistent condom use were common among this sample. CCLs, which decrease the risk of condom breakage, were also used inconsistently [33], suggesting that increased access to quality condoms and CCLs is essential [34,35]. While maximizing the use of condoms and CCLs is necessary in decreasing HIV risks among MSM, likely it will not be sufficient to change the trajectory of the epidemic given the high transmission probability of HIV infection associated with UAI, as observed in other settings [1,34]. The prevalence of active syphilis was low, as observed in other countries in the region [4,5]; however, a high proportion of participants reported experiencing STI symptoms, highlighting another network-level risk factor potentiating HIV transmission within the sexual network. Increasing the capacity for routine STI diagnosis, particularly for genitourinary infections, and linkage to treatment tailored towards MSM should be incorporated to support HIV-prevention programmes [15,36].

MSM in Douala who reported a preference of being the receptive partner during anal intercourse were more likely to identify as gay and be living with HIV. This not only affirms existing data demonstrating the increased HIV acquisition risk associated with unprotected receptive anal intercourse [37] but also echoes previous studies conducted in African settings in which self-reporting as gay was associated with higher odds of living with HIV compared to other MSM in the African setting [24,38]. Given that antiretroviral pre-exposure prophylaxis (PrEP) and rectal microbicides have been identified as research priorities for African MSM [39], and that rectal microbicides are currently in Phase II trials that are enrolling MSM from the African continent [34], evaluating the feasibility of novel biomedical interventions for sub-populations of MSM in Cameroon with significant HIV acquisition risks may be appropriate [34,40,41]. However, the cost-effectiveness of implementationing such biomedical interventions requires further research [42]. In addition, exploring increased antiretroviral therapy (ART) for MSM living with HIV likely represents an important strategy for preventing the transmission of HIV to sexual partners. However, the limited availability of ART for people living with HIV who are currently eligible for treatment, which has been documented in Cameroon, also needs to be addressed in order for ART-based strategies for people at risk for the acquisition or transmission of HIV to be effective [43].

A significant proportion of the MSM in our sample were living with HIV by the age of 18–23, indicating a high risk for HIV acquisition for men under 18 in these settings [24,44]; however, men under 18 have traditionally been excluded from HIV surveillance and prevention programmes [1]. Confidential youth sexuality counselling hotlines, web-based education and social marketing campaigns may be useful in reaching younger MSM with HIV programmes [36,37].

While our study did not include a detailed assessment of social stigma, other studies have demonstrated that stigma limits the provision and uptake of HIV prevention, treatment and care for MSM in the region [18,19,27]. Uptake of services delivered by targeted CBO providers such as Alternatives-Cameroun in Douala was high in our study, suggesting that community-based approaches can spread information-leveraging networks of MSM despite the contextual barriers. There was limited uptake of services in Yaoundé and higher refusal of HIV testing in the study; to the best of our knowledge, MSM-tailored HIV programmes were new and in development at the time of this surveillance project. The historically limited services may partially explain the higher HIV prevalence observed among MSM in Yaoundé as compared to Douala, although these participants also tended to be older and report more male partners, drug and alcohol use, and STI symptoms.

Data on the proportion of MSM living with HIV who were eligible for treatment, or who were actually on treatment, were not collected in this study. However, consistent data highlight the importance of addressing the needs of people living with HIV, including linkage to care, to optimize their own health and prevent onward transmission to other men and to women [45]. In Cameroon, only half of all patients eligible for treatment are estimated to be receiving ART, and ART stock outages at health facilities are frequent [43]. Given the significant stigma and discrimination that have been documented as affecting MSM in Cameroon, MSM living with HIV may be at higher risk of being unaware of their diagnosis or not achieving viral suppression [8,20,46]. MSM community groups have long been known to play essential roles in the HIV response, and the data collected here suggest that community-driven approaches should be scaled up to increase uptake of VCT and support linkage to HIV care, treatment and adherence support for those eligible [47,48].

The cross-sectional design of this study does not allow us to assume causality of the associations present in the data. There are several limitations to the generalizability of the HIV prevalence estimates reported in this study, which included individuals who reported receptive or insertive anal intercourse in the past 12 months. The generalizability of the results for MSM living in smaller urban centres and rural settings is unknown given that recruitment occurred in two large cities. Similarly, as our sample was predominantly young and educated, the results may not pertain to older MSM or individuals with lower educational status. Future studies to address these gaps could be conducted. The modest sample size may have reduced our statistical ability to detect other associations [49]. Due to the high refusal of HIV testing during the study in Yaoundé (13.4%), we were unable to assess the potential for bias in the HIV prevalence estimate from this city. However, RDS network homophily was close to 0, which may indicate minimal recruitment bias based on HIV status. Data on self-reported HIV status and the percentage of undiagnosed men were not available, which limit our interpretation of the association between knowledge of one's own HIV status and behavioural factors such as inconsistent use of condoms and CCLs. This requires further investigation in future studies. Although non-significant, the positive association between having been tested and refusing testing may suggest that individuals who are already aware of their HIV status may be underrepresented in our study.

## Conclusions

These data provide results that can be integrated into HIV programmes for MSM in Cameroon and highlight the importance of targeted HIV prevention, treatment and care services that address all levels of HIV risk. Coordinating behavioural, biomedical and structural interventions, and supporting the work of local CBOs, will be keys to ensuring that HIV-negative MSM receive regular VCT and appropriate prevention services, and that MSM living with HIV are effectively engaged in the continuum of HIV care. Success in the continuum of HIV care necessitates addressing the barriers to the uptake of care, such as concerns about confidentiality and healthcare-related enacted and perceived stigmas [8,20,36]. Protecting the dignity and rights of MSM in healthcare settings and beyond allows for a safe environment for individuals to receive optimal care to protect themselves and their partners [27,29]. Monitoring the success of the next generation of HIV-prevention approaches will require innovative implementation science exploring changes not only in individual-level risks, community viral load and HIV incidence, but also in social and policy-level factors including stigma, discrimination, violence and criminalization.
